# Crystal Structures of Antigen-Binding Fragment of Anti-Osteocalcin Antibody KTM219

**DOI:** 10.3390/ijms26020648

**Published:** 2025-01-14

**Authors:** Shuma Yazaki, Misaki Komatsu, Jinhua Dong, Hiroshi Ueda, Ryoichi Arai

**Affiliations:** 1 Department of Applied Biology, Faculty of Textile Science and Technology, Shinshu University, Ueda 386-8567, Nagano, Japan; 2 Laboratory for Chemistry and Life Science, Institute of Innovative Research, Tokyo Institute of Technology, Yokohama 226-8503, Kanagawa, Japan; jhdong@uor.edu.cn (J.D.);; 3 Department of Biomolecular Innovation, Institute for Biomedical Sciences, Interdisciplinary Cluster for Cutting Edge Research, Shinshu University, Ueda 386-8567, Nagano, Japan

**Keywords:** antibody, antigen–antibody complex, biosensor, crystal structure, immunoassay, immunosensor, fluorescence, osteocalcin, Quenchbody (Q-body)

## Abstract

Osteocalcin is a useful biomarker for bone formation and bone-related diseases. KTM219 is an anti-osteocalcin C-terminal peptide antibody. The single-chain variable region (scFv) and antigen-binding fragment (Fab) of KTM219 are applicable to the Quenchbody (Q-body) immunoassay. Q-body is a new type of fluorescent immunosensor, which is scFv or Fab labeled with a fluorescent dye. When Q-body binds to its antigen, the fluorescence intensity increases. The highly sensitive detection of antigens by changes in fluorescence intensity is performed in a single step by mixing the sample and reagent. In this study, to reveal the recognition mechanism of the KTM219 antibody and to discuss the structural basis for Q-body, we solved the crystal structures of Fab of the anti-osteocalcin antibody KTM219 and its complex with the antigen osteocalcin C-terminal peptide (BGP-C7). Also, we solved the structure of a KTM219 Fab crystal grown in the presence of a fluorescent dye, carboxytetramethylrhodamine (TAMRA); however, tightly bound TAMRA was not found in the electron density map. We predicted the binding sites of TAMRA in the antigen-binding pocket by docking simulations. These results support the proposed Q-body mechanism. The crystal structures of KTM219 Fab would be useful for further development and improvement of Q-body fluorescent immunosensors.

## 1. Introduction

Immunoassays, analytical methods using antibodies, have become increasingly popular in diagnostics and clinical research to detect various biomarkers, antigens, and antibodies with high sensitivity and specificity [1,2,3]. However, conventional immunoassays, such as an enzyme-linked immunosorbent assay (ELISA), are relatively time-consuming. Although faster and more convenient methods such as immunochromatography have been widely used, they are generally not very quantitative and require more than several minutes to complete the measurement with sufficient sensitivity. To overcome such problems, homogeneous immunosensors based on fluorescence or bioluminescence have been developed using antibody engineering [4]. Fluorescent immunosensors have many advantages, such as fast response, high sensitivity and selectivity compared to colorimetric and absorbance-based analyses, and easy labeling due to many available fluorescent reagents. For example, Förster resonance energy transfer (FRET)-based immunosensors have been developed as a type of biosensor to detect and quantify specific proteins or other biomolecules [4,5]. FRET is a non-radiative energy transfer between two fluorophores from an excited donor to a nearby acceptor.

As a leading example of fluorescent immunosensors with a single fluorophore, Quenchbody (Q-body) has been developed [6,7,8,9]. Q-body is a fluoro-labeled antibody, single-chain variable region (scFv) or antigen-binding fragment (Fab), and its fluorescence intensity increases when its antigen is added. At the beginning of Q-body development, carboxytetramethylrhodamine (TAMRA)-labeled scFvs were prepared using a position-specific protein-labeling method based on fluoro-labeled aminoacyl tRNA and a cell-free translation system [6]. An anti-osteocalcin C-terminal peptide antibody, KTM219 [10] is one of antibodies applicable to the Q-body immunoassay [6].

However, the stability and affinity of scFv in comparison with its parental full-size antibody or Fab have been a problem [11]. To explore the practical utility of Q-body, Fab-based Q-bodies (also called Ultra-Quenchbodies (UQ-bodies)) were developed [7]. Fab fragments were fluoro-labeled at either one or two of the N-terminal regions, using a cell-free translation-mediated position-specific protein-labeling system. In addition, a Fab-based Q-body was successfully produced using a combination of recombinant protein expression in *Escherichia coli* and thiol-based fluorescence labeling [7].

With the homogeneous assay using Q-body, a positive fluorescence signal can be readily obtained within a few minutes depending on the antigen concentration in a sample. In contrast to ELISA, the Q-body assay does not require time-consuming procedures such as washing and enzyme reaction steps. In addition to proteins and peptides, small molecules with molecular weights below ~1000 Da, such as morphine and estradiol, can also be specifically detected with similar sensitivity in competitive assays [6,12,13,14].

Regarding the mechanism of action of Q-body, a model based on conformational change upon antigen binding has been proposed [6,8]. In the absence of antigen, a fluorescent dye such as 5(6)-carboxytetramethylrhodamine (TAMRA), attached to the N-terminus of scFv or Fab through a short linker, transiently interacts with the highly conserved tryptophan (Trp) residues in the antigen-binding region. Mutagenesis of the Trp residues resulted in the attenuation of fluorescence quenching, suggesting that the Trp residues are important for the quenching process [6], probably due to photoinduced electron transfer [15,16]. In the presence of antigen, V_H_ and V_L_ interact strongly [10], and the dye competitively moves out of the variable region (Fv); therefore, the quenching effect is reduced depending on the antigen concentration, resulting in increased fluorescence. In addition, the mechanism of Q-body was supported by analysis of the dynamics of dye movement in Q-body using ELISA and fluorescence polarization assay [17]. However, detailed structural information is needed for further understanding.

Human osteocalcin (bone Gla protein, BGP) is a useful biomarker for bone formation and bone-related diseases [18]. The anti-osteocalcin C-terminal peptide antibody KTM219 [10] is a representative Fab fragment applicable to the Q-body immunoassay [7]. KTM219 Fab with one or two Cys-containing peptide tag(s) at the N-terminal region(s) was expressed in *E. coli*. After protein purification, KTM219 Fab was labeled with a thiol-reactive fluorescent dye such as TAMRA-C5-maleimide. A significant antigen-dependent increase in fluorescence was observed for the single TAMRA-labeled Q-body (labeled at the N-terminus of V_H_), which showed a 7.0-fold increase in fluorescence with a low EC_50_ of 1.9 × 10^−8^ M. In contrast, the double TAMRA-labeled Q-body (labeled at the N-termini of V_H_ and V_L_) showed a relatively modest antigen-dependent response of up to 2.4-fold [7]. Hereafter, we focus on the single TAMRA-labeled Fab-based Q-body (KTM219 Fab fluoro-labeled at N-terminus of V_H_) to discuss the mechanism of Q-body.

In this study, to reveal the recognition mechanism of the KTM219 antibody and to discuss the structural basis for Q-body, we report here the crystal structures of KTM219 Fab.

## 2. Results

### 2.1. Crystal Structure of KTM219 Fab

**Figure 1:**
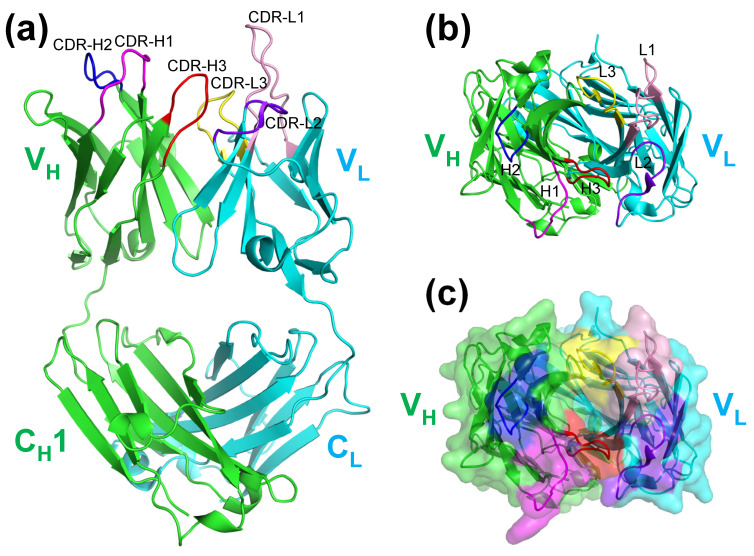
Crystal structure of KTM219 antigen-binding fragment (Fab). (**a**) Ribbon representation of the overall structure (Protein Data Bank (PDB) ID: 5X5X). (**b**) Top view of ribbon representation. (**c**) Top view of surface representation. A deep pocket between V_H_ and V_L_ suggests a putative antigen-binding site. The heavy and light chains are shown in green and cyan, respectively. Complementarity-determining regions (CDRs) are shown in different colors (CDR-H1, magenta; CDR-H2, blue; CDR-H3, red; CDR-L1, light pink; CDR-L2, purple blue; CDR-L3, yellow).

First, we solved the crystal structure of the Fab antibody fragment of the anti-osteocalcin antibody KTM219. The KTM219 Fab crystal belonged to the orthorhombic space group *P*2_1_22_1_, with unit cell constants of *a* = 64.80 Å, *b* = 71.47 Å, and *c* = 96.88 Å and contained one Fab antibody fragment per asymmetric unit. The structure was solved by the molecular replacement method with a model structure of anti-emmprin antibody 4A5 Fab (Protein Data Bank (PDB) ID: 4KUZ) [19]. The structure was refined to 1.90 Å resolution (*R*_work_ = 18.1%, *R*_free_ = 22.4%). All refinement statistics are shown in Table S1. The crystal structure of KTM219 Fab (PDB ID: 5X5X) comprises a light chain (V_L_–C_L_) and a heavy chain (V_H_–C_H_1) with typical immunoglobulin folds with complementarity-determining regions (CDRs) (Figure 1a). A deep pocket is formed between V_H_ and V_L_, and it can provide a putative binding site for the antigen (Figure 1b,c).

### 2.2. Crystal Structure of Antibody–Antigen Complex of KTM219 Fab

**Figure 2:**
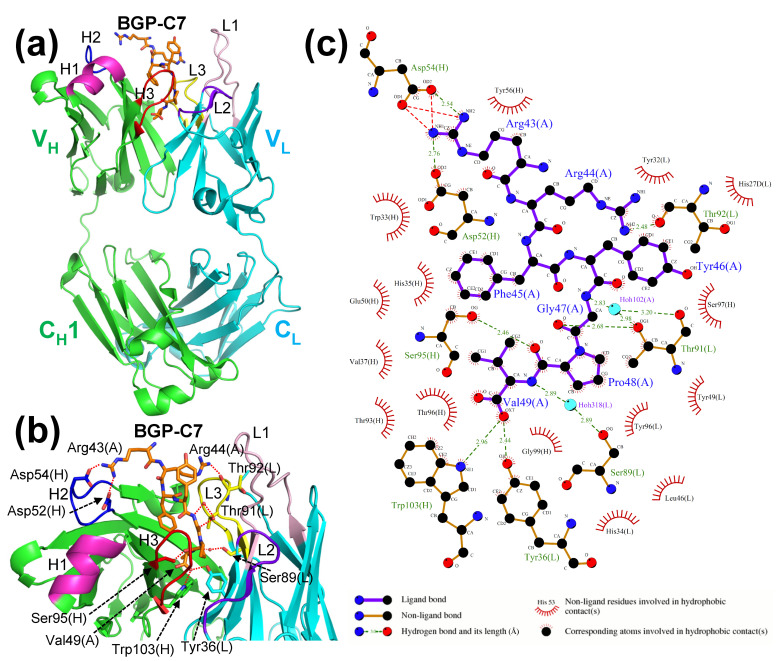
Crystal structure of antibody–antigen complex of KTM219 Fab. (**a**) Ribbon representation of the overall structure (PDB ID: 8XS1). The heavy and light chains are shown in green and cyan, respectively. The antigen peptide BGP-C7 (RRFYGPV) is shown as orange sticks. CDRs are shown in different colors (CDR-H1, magenta; CDR-H2, blue; CDR-H3, red; CDR-L1, light pink; CDR-L2, purple blue; CDR-L3, yellow). (**b**) Closeup view of the binding site of the antibody–antigen complex. Important residues for the antibody–antigen interactions with hydrogen bonds (red dashed lines) are shown as sticks. (**c**) Antibody–antigen interaction diagrams generated using LigPlot^+^ [20]. (A), (H) and (L) after residue numbers represent antigen, H chain and L chain of antibody, respectively.

To reveal the recognition mechanism of the KTM219 antibody and antigen, we crystalized the antibody–antigen complex of KTM219 Fab and osteocalcin C-terminal 7-residue peptide BGP-C7 (RRFYGPV). The crystal of the complex belonged to the orthorhombic space group *P*2_1_2_1_2_1_, with unit cell constants of *a* = 43.88 Å, *b* = 67.49 Å, and *c* = 138.66 Å and contained one complex of the KTM219 Fab antibody and the BGP-C7 antigen per asymmetric unit. The structure was refined to 2.30 Å resolution (*R*_work_ = 19.9%, *R*_free_ = 24.8%). The crystal structure (PDB ID: 8XS1) clearly shows the binding site of the antigen BGP-C7 in detail (Figure 2). The BGP-C7 peptide binds to the hydrophobic pocket between V_H_ and V_L_. The C-terminal residue Val49(A) of the antigen peptide is buried in the deep hydrophobic pocket. The details of the interactions of antibody and antigen are shown in Figure 2c depicted using LigPlot^+^ [20]. The antigen peptide residues are recognized by several hydrogen bonds, many hydrophobic interactions, and a few salt bridges. Specifically, the terminal carboxy group of antigen Val49(A) is recognized by hydrogen bonds from the antibody residues Trp103(H) and Tyr36(L) (according to the Kabat numbering scheme [21]). In addition, two waters mediate the interactions between amide protons of the antigen and Ser89(L) and Thr91(L) in CDR-L3 of the antibody. Moreover, the salt bridges between the antigen residue Arg43(A) and the antibody residues Asp52(H) and Asp54(H) in CDR-H2 contribute to antibody–antigen recognition. These findings are fully consistent with the experimental results of the binding assay by open sandwich ELISA [10]. C-terminal 10-residue peptide BGP-C10 (EAYRRFYGPV), 8-residue peptide BGP-C8 (YRRFYGPV), and 7-residue peptide BGP-C7 (RRFYGPV) showed full binding activities. In contrast, 9-residue peptide BGP-C10dV (C10 without C-terminal Val, EAYRRFYGP) and C-terminal 5-residue peptide BGP-C5 (FYGPV) showed negligible binding, and C-terminal 6-residue peptide BGP-C6 (RFYGPV) showed much less binding than BGP-C7.

### 2.3. Structure of KTM219 Fab Crystal Grown in the Presence of a Fluorescent Dye TAMRA

To elucidate the mechanism of Q-body, we tried to crystalize Cys-tagged KTM219 Fab (Cys-containing peptide tag (MSKQIEVNYCSNETG) was added to N-terminus of V_H_ [7]) chemically modified with TAMRA-C5-maleimide (Biotium, Hayward, CA, USA) using thiol target labeling. However, we did not obtain crystals suitable for structural analysis.

**Figure 3:**
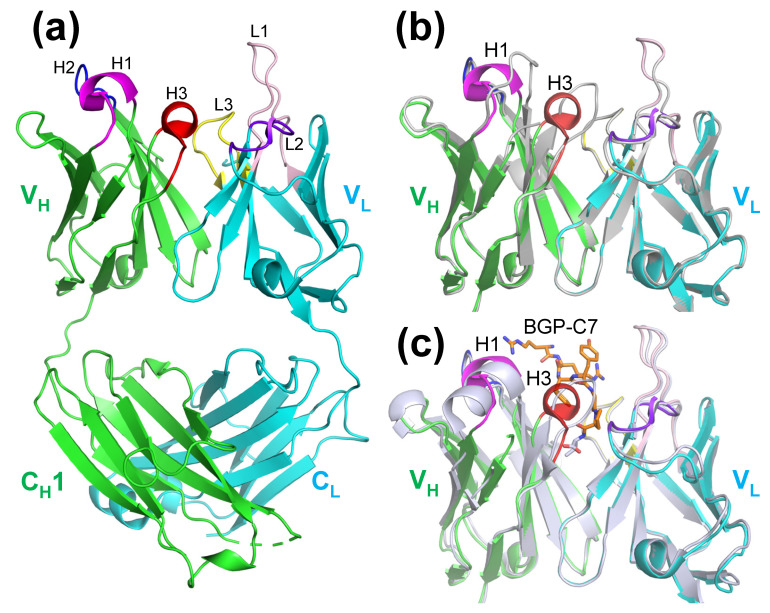
Structure of the KTM219 Fab crystal grown in the presence of the free fluorescent dye TAMRA. (**a**) Ribbon representation of the overall structure (PDB ID: 8XS2). The heavy and light chains are shown in green and cyan, respectively. CDRs are shown in different colors (CDR-H1, magenta; CDR-H2, blue; CDR-H3, red; CDR-L1, light pink; CDR-L2, purple blue; CDR-L3, yellow). (**b**) Structural alignment of main-chain structures of KTM219 variable region (Fv) (green/cyan) with the addition of TAMRA (PDB ID: 8XS2) and KTM219 Fv (gray) without the addition of TAMRA (PDB ID: 5X5X). (**c**) Structural alignment of main-chain structures of KTM219 Fv (green/cyan) with the addition of TAMRA (PDB ID: 8XS2) and KTM219 Fv (light gray) complexed with the antigen (PDB ID: 8XS1). The antigen peptide BGP-C7 is shown as orange sticks.

Therefore, we grew the KTM219 Fab crystal in the presence of free TAMRA fluorophore. The crystal belongs to the orthorhombic space group *P*2_1_22_1_, with unit cell constants of *a* = 66.19 Å, *b* = 69.52 Å, and *c* = 96.65 Å and contains one Fab antibody fragment per asymmetric unit. The structure (PDB ID: 8XS2) was refined to 2.14 Å resolution (*R*_work_ = 20.2%, *R*_free_ = 24.4%) (Figure 3a). Although the overall structure is almost the same with/without the addition of TAMRA, the CDR-H1 and CDR-H3 loops of V_H_ near the binding pocket have different conformations (Figure 3b). However, we did not find the electron density assigned to TAMRA. A detailed comparison of the structure with the addition of TAMRA and the structure complexed with the antigen shows that the CDR-H3 loop has different conformations, although the overall structure is almost identical (Figure 3c). In addition, the CDR-H1 structure with the addition of TAMRA has a partial helix, which partly resembles a helical conformation of CDR-H1 in the antibody–antigen complex (Figure 3c). These findings suggest that TAMRA does not tightly bind to the specific site on the antibody and that weak interactions with TAMRA may affect the structural conformations of the CDR loops of the antibody around the antigen-binding pocket.

### 2.4. Docking Simulations of Fluorescent Dye TAMRA to KTM219 Fab

To predict the Q-body structure complexed with the fluorescent dye TAMRA, we performed docking simulations of TAMRA to the KTM219 Fab structure of the crystal grown in the presence of TAMRA (PDB ID: 8XS2).

**Figure 4:**
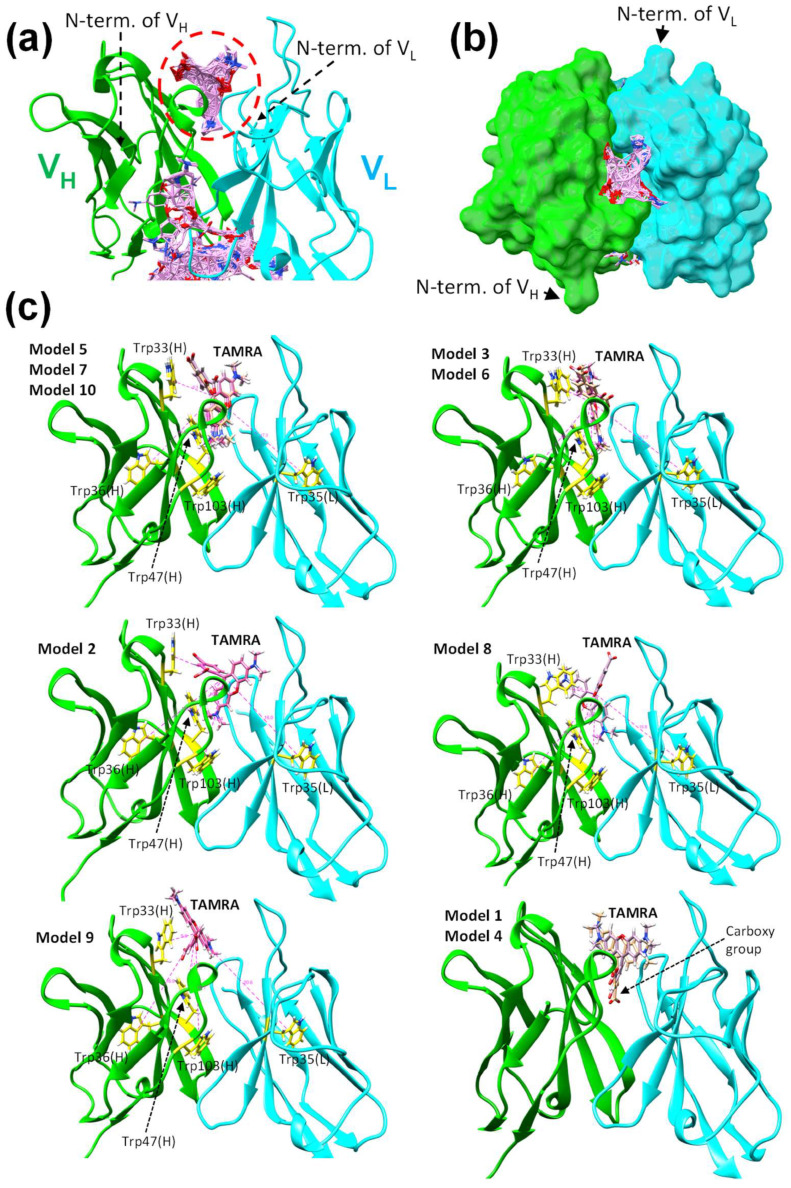
Docking simulations of carboxytetramethylrhodamine (TAMRA) to KTM219 Fab. (**a**,**b**) Docking simulation results using SwissDock. (**a**) Closeup view of predicted interaction sites at the antigen-binding pocket near the N-terminus. Docking poses of TAMRA are shown as pink sticks. A dashed red circle indicates dense clusters of docking poses in the antigen-binding pocket. Overall view is shown in Figure S1. (**b**) Top view of predicted interaction sites at the antigen-binding pocket of the antibody (surface representation). (**c**) Docking simulation results using RosettaLigand in ROSIE. The top 10 models with low interface scores (i.e., good docking models) were selected and divided into six classes based on orientations of TAMRA. Tryptophan (Trp) residues (Trp33(H), Trp36(H), Trp47(H), Trp103(H), and Trp35(L)) are shown as yellow sticks. Values with dashed pink lines show distances between TAMRA and Trp residues. The distances between TAMRA and Trp residues are summarized in Table S2. The antibody domains V_H_ and V_L_ are shown in green and cyan, respectively.

First, we used SwissDock [22] based on EADock DSS [23] to search for possible interaction sites of TAMRA to the KTM219 Fab structure. The whole target protein structure of the KTM219 Fab was considered to search for possible binding pockets during the docking. Several clusters of predicted docking poses (256 poses in total) are shown on the protein structure (Figure S1), suggesting several candidates for interaction sites. In particular, 24 poses formed dense clusters in the antigen-binding pocket (Figure 4a,b). Hereafter, we focused on the antigen-binding pocket near the N-terminus of V_H_ as a major interaction site because we focused on the single TAMRA-labeled Q-body (KTM219 Fab fluoro-labeled at the N-terminus of V_H_) in this study.

To predict detailed conformations of TAMRA bound to the antigen-binding pocket, we performed docking simulations using RosettaLigand in the Rosetta Online Server that Includes Everyone (ROSIE) [24]. The central part of the antigen-binding pocket was set as an initial position of TAMRA. From the results (200 models) of RosettaLigand docking simulations (Figure S2), the top 10 models with lower interface scores (i.e., better docking models) were selected. The two models (Model 1 and Model 4) with unrealistic conformations were omitted because their carboxy group, which should be linked to a maleimide linker, buried in the deep pocket could not lead to the N-terminus of V_H_ with a maleimide linker due to the limited space (Figure 4c). The eight models with realistic conformations were selected and further divided into five classes based on the orientations of TAMRA (Figure 4c). These docking simulation results suggest that TAMRA can bind to the antigen-binding pocket of KTM219 Fab with several conformations. The interaction residues with TAMRA are shown in Figure S3a, depicted using LigPlot^+^ [20] in three typical models (Model 5, Model 3, and Model 2). Several hydrogen bonds and hydrophobic interactions were predicted to contribute to the binding between antibody and TAMRA. In addition, tryptophan residues, Trp33(H), Trp36(H), Trp47(H), Trp103(H), and Trp35(L), are located around the predicted binding sites of TAMRA (Figure 4c) in distances of ~5–20 Å (Table S2). Moreover, the binding sites of TAMRA and the antigen BGP-C7 are significantly overlapped (Figure S3b). These simulation results and the crystal structure of the antibody–antigen complex suggest the molecular competition between the fluorescent dye and the antigen to occupy the shared binding site.

## 3. Discussion

**Figure 5:**
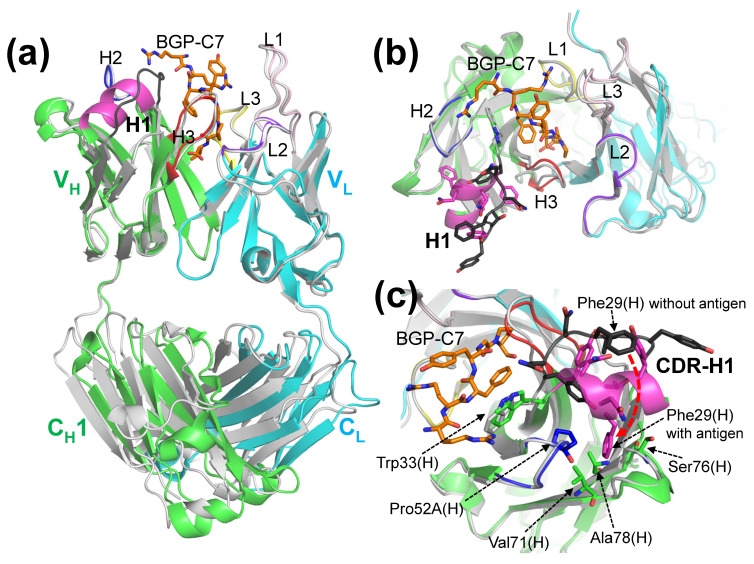
Comparisons of the KTM219 Fab structures with/without the antigen. (**a**) The superimposition of the main-chain structures (ribbon representation) aligned in the V_H_ and V_L_ domains. The heavy and light chains of the KTM219 Fab structure with the antigen (PDB ID: 8XS1) are shown in green and cyan, respectively. The antigen peptide BGP-C7 (RRFYGPV) is shown as orange sticks. CDRs are shown in different colors (CDR-H1, magenta; CDR-H2, blue; CDR-H3, red; CDR-L1, light pink; CDR-L2, purple blue; CDR-L3, yellow). The KTM219 Fab structure without antigen (PDB ID: 5X5X), except the CDR-H1 loop, is shown in light gray. The CDR-H1 structure without the antigen is shown in dark gray. (**b**) Closeup view of the antigen-binding site. The residues of CDR-H1 (GYTFNNY) are shown as sticks. (**c**) Closeup view of the CDR-H1 regions. The residues remarked in the text are labeled and shown as sticks. A dashed red arrow represents an expected movement of Phe29(H) upon antigen binding.

We solved the crystal structures of the anti-osteocalcin antibody KTM219 Fab and its complex with the antigen BGP-C7. Figure 5 shows a comparison of the two structures. Although the overall structures of V_H_ and V_L_ with/without the antigen are almost identical, the structures of CDR-H1 have significantly different conformations (Figure 5a). The structure of the CDR-H1 loop was changed to a short helix upon antigen binding, although the residues of CDR-H1 did not interact directly with the antigen peptide (Figure 5b). When the antigen binds to the antibody and then Trp33(H) is slightly shifted, this structural change is probably induced by the insertion of the side chain of Phe29(H) into a hydrophobic pocket due to hydrophobic interactions of Phe29(H) with Pro52A(H), Val71(H), Ser76(H), and Ala78(H) (Figure 5c).

**Figure 6:**
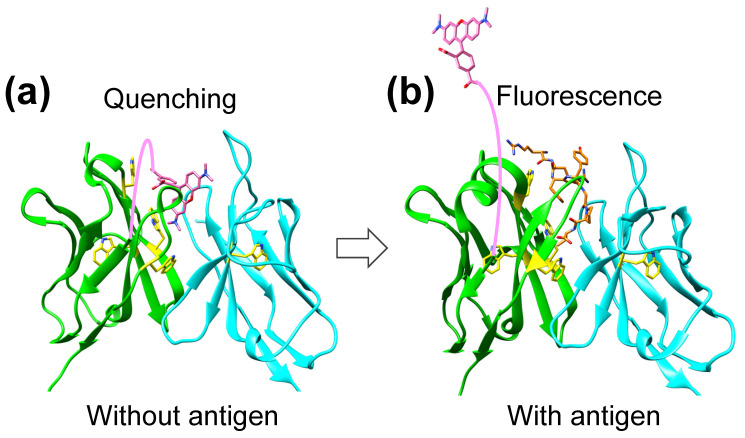
Diagrams of structural basis for the Q-body mechanism based on the crystal structures. (**a**) Without an antigen, a quenched fluorophore is located near the Trp residues (Trp33(H), Trp36(H), Trp47(H), Trp103(H), and Trp35(L)) in the hydrophobic pocket of the antigen-binding site between V_H_ and V_L_. The V_H_ and V_L_ domains are shown in green and cyan, respectively (PDB ID: 8XS2). The fluorophore TAMRA is shown as pink sticks. Trp residues are shown in yellow sticks. A pink line represents a linker between TAMRA and N-terminus of V_H_ (N-terminal Cys-tag chemically fluoro-labeled with TAMRA-C5-maleimide). (**b**) In association with the competitive binding of the antigen, the fluorophore TAMRA is released from the antigen-binding pocket and emits fluorescence. The V_H_ and V_L_ domains are shown in green and cyan, respectively (PDB ID: 8XS1). The BGP-C7 antigen peptide is shown as orange sticks.

Q-body is an antibody-based immunosensor constructed by labeling the N-terminal region of scFv or Fab of an antibody with a fluorescent dye [6,7,8]. This technique is easy to operate by simply adding the sample to the Q-body reagent solution and measuring the fluorescence intensity within a few minutes. The following putative mechanism of Q-body has been proposed [6,8]. In the absence of the antigen, the fluorescent dye enters the hydrophobic interface region between V_H_ and V_L_ due to the hydrophobicity of the dye; the fluorophore is quenched by Trp residues in the hydrophobic pocket near the antigen-binding site between V_H_ and V_L_. When Q-body binds to the antigen, the dye moves outward due to the antigen-dependent Fv stabilization and steric hindrance by the competitively bound antigen and recovers its fluorescence by dequenching. Thus, the antigen concentration can be detected by measuring the positive change in fluorescence intensity. Moreover, analyses of the dynamics of dye movement in the reaction between Q-body and antigen by ELISA and fluorescence polarization experiments have supported the proposed mechanism of Q-body [17]. The proposed mechanism of Q-body is clearly supported by the crystal structures of KTM219 Fab (Figure 6). In addition, in the structural analysis of the KTM219 Fab crystal grown in the presence of TAMRA, the electron density assigned to TAMRA could not be found, suggesting weak and dynamic interactions between the antibody and TAMRA. It may be reasonable that TAMRA is easily replaced by the antigen due to the weak and dynamic interactions between the antibody and TAMRA, contributing to high sensitivity of the Q-body immunoassay.

Specific amino acids, namely several Trp residues in the antibody variable region attenuate the fluorescence of the dye by quenching due to photoinduced electron transfer from Trp to the dye [6]. Tryptophan with an indole side chain is an effective electron donor in the reaction with dye molecules because it is one of the most easily oxidized functional groups among natural amino acids [15,16]. Antibody fragments quench the coupled fluorescent dye through the Trp residues semiconserved in its variable region. The crystal structure of KTM219 Fab and the docking simulations with TAMRA reveal that there are the five Trp residues (Trp33(H), Trp36(H), Trp47(H), Trp103(H), and Trp35(L)) around the antigen-binding pocket with appropriate distances (Table S2) from the predicted binding sites of TAMRA for quenching by photoinduced electron transfer [16,25]. In addition, Trp33(H) and Trp103(H) interact directly with the antigen peptide BGP-C7 according to the crystal structure of the antibody–antigen complex (Figure 2c).

In a previous study, Q-bodies with single point mutations of these five Trp to Phe were prepared, and the antigen-dependent fluorescence intensities were measured [6]. All mutant Q-bodies showed attenuated changes in fluorescence intensity upon addition of the antigen peptide BGP-C7, when compared to the wild-type Q-body. Compared with the basal intensity without antigen, the fluorescence enhancements observed upon addition of antigen were 3.7-, 4.7-, 2.5-, 3.5-, and 3.1-fold for the W33F(H), W36F(H), W47F(H), W103F(H), and W35F(L) mutants, respectively. These values are lower than the value observed for the wild-type scFv (5.6-fold), suggesting that the five Trp residues are involved in the Q-body mechanism. These results are basically consistent with the results from the crystal structures and docking simulations. In addition, the four Trp residues (Trp36(H), Trp47(H), Trp103(H), and Trp35(L)) are located in the framework region of Fv and are highly conserved (>93%) among immunoglobulins of various origins, suggesting the potential generality of the Q-body methodology [6,8].

In this study, the crystal structures of KTM219 Fab and the antibody–antigen complex reveal the antibody–antigen recognition mechanism. Furthermore, the structures and docking simulations with a fluorescent dye (TAMRA) clearly support the proposed mechanism of Q-body, which is summarized as the molecular competition between the antibody-tethered fluorescent dye and the antigen in a sample (Figure 6). These structural analyses provide the structural basis for Q-body, and these findings would be useful for further development and improvement of Q-body fluorescent immunosensors.

## 4. Materials and Methods

### 4.1. Protein Expression and Purification

The Fab fragment of anti-osteocalcin C-terminal peptide antibody KTM219 was expressed in *E. coli* SHuffle T7 Express *lysY* (New England BioLabs, Ipswich, MA, USA) with a protein expression plasmid pET-Fab (KTM219) [26] as previously described [7,26]. The His_6_-tagged proteins were purified using immobilized metal affinity chromatography with TALON Metal Affinity Resin (Takara Bio, Kusatsu, Shiga, Japan). The equilibration/wash buffer contained 50 mM sodium phosphate buffer (pH 7.0) and 300 mM NaCl, and the elution buffer contained 50 mM sodium phosphate buffer (pH 7.0), 300 mM NaCl, and 150 mM imidazole. The protein was further purified by anion exchange chromatography (20 mM Tris-HCl buffer (pH 8.5) with a linear gradient of NaCl from 0 to 1 M) with a RESOURCE Q 1 mL column (Cytiva, Little Chalfont, Buckinghamshire, UK) and size-exclusion chromatography (20 mM Tris-HCl buffer (pH 8.0) containing 150 mM NaCl) with a Superdex 75 10/300 GL column (Cytiva). The Fab fragment of KTM219 with the addition of three molar excess BGP-C7 (NH_2_-RRFYGPV-COOH) was also purified by gel filtration chromatography to obtain the antibody–antigen complex.

### 4.2. Crystallization

The KTM219 Fab protein (4 mg/mL) was crystallized at 20 °C using the hanging drop vapor diffusion method. The KTM219 Fab (1 μL) was mixed with the same volume of reservoir solution (0.6 M sodium formate, 29% *w*/*v* polyethylene glycol (PEG) 3350).

The antibody–antigen complex (KTM219 Fab + BGP-C7) was crystallized at 20 °C using the sitting and hanging drop vapor diffusion methods. Microcrystals and polycrystals were obtained in several conditions, such as reservoir solution (0.2 M sodium formate, 20–35% *w*/*v* PEG 3350). Furthermore, to obtain better crystals, we used a random microseed matrix screening (rMMS) method, a crystal seeding method with random screening kits [27]. The protein complex (15 mg/mL) and reservoir solution were mixed with microseeds prepared from the microcrystals using Seed Bead (Hampton Research, Aliso Viejo, CA, USA). The best crystal for X-ray crystallography was obtained with a reservoir solution (0.07 M citric acid, 0.03 M Bis-tris propane (pH 3.4), 16% *w*/*v* PEG 3350) using the sitting drop vapor diffusion method.

The KTM219 Fab protein (6 mg/mL) with the addition of five molar excess TAMRA (Biotium, Hayward, CA, USA; The stock solution was dissolved in dimethyl sulfoxide.) was mixed with the same volume of reservoir solution (0.3 M ammonium acetate, 0.1 M HEPES (pH 7.5), 25% *w*/*v* PEG 3350). The crystal of the KTM219 Fab protein was grown in the presence of TAMRA at 20 °C using the hanging drop vapor diffusion method.

### 4.3. Data Collection, Structure Determination, and Refinement

X-ray diffraction data were collected using the KEK Photon Factory (PF) Structural Biology Beamline BL-5A or AR-NW12A at 95 K with reservoir solution added to 25% *w*/*v* PEG 400 or 25% *w*/*v* glycerol as a cryoprotectant. The diffraction data were processed with the program HKL2000 [28]. The structure was solved by molecular replacement method using Phaser with a model structure of anti-emmprin antibody 4A5 Fab (PDB ID: 4KUZ) [19]. The model was corrected with the program COOT ver. 0.8.2 [29] and was refined with the program REFMAC ver. 5.8 [30] in the CCP4 suite [31]. The quality of the model was inspected by the programs PROCHECK [32], RAMPAGE [33], and MolProbity [34]. All data collection and refinement statistics are shown in Table S1. The atomic coordinates and the structure factors have been deposited in PDB with the accession codes 5X5X (KTM219 Fab), 8XS1 (KTM219 Fab + BGP-C7), and 8XS2 (KTM219 Fab + TAMRA). The graphic figures were created using the programs open-source PyMOL ver. 2.4 (Schrödinger, New York, NY, USA), UCSF Chimera ver. 1.13 [35], and UCSF ChimeraX ver. 1.6 [36].

### 4.4. Docking Simulations

To predict the possible interaction sites of TAMRA to the KTM219 Fab structure, docking simulations of the fluorescent dye TAMRA to the KTM219 Fab structure of the crystal grown in the presence of TAMRA (PDB ID: 8XS2) were performed using SwissDock [22] based on EADock DSS [23]. The whole target protein structure of KTM219 Fab was considered during the docking.

In addition, to predict the detailed conformations of TAMRA bound to the antigen-binding pocket of KTM219 Fab, docking simulations were performed using RosettaLigand [37,38] in ROSIE [24]. The initial position of the fluorescent dye TAMRA was set at the central part of the antigen-binding pocket of KTM219 Fab.

## 5. Conclusions

In this study, we solved the crystal structures of the anti-osteocalcin antibody KTM219 Fab and its complex with the antigen BGP-C7, revealing the antibody–antigen recognition mechanism. In addition, we predicted the binding sites of TAMRA in the antigen-binding pocket by docking simulations. These results clearly support the proposed mechanism of Q-body as follows. In the absence of antigen, the fluorophore is located near the tryptophan residues (Trp33(H), Trp36(H), Trp47(H), Trp103(H), and Trp35(L)) in the hydrophobic pocket of the antigen-binding site between V_H_ and V_L_ and is quenched by photoinduced electron transfer. In association with the competitive binding of the antigen, the fluorophore is released from the antigen-binding pocket and emits fluorescence due to dequenching. The crystal structures of KTM219 Fab and discussion on the structural basis for Q-body would be useful for further development and improvement of Q-body fluorescent immunosensors.

## Data Availability

The atomic coordinates and the structure factors have been deposited in the Protein Data Bank with the accession codes 5X5X, 8XS1, and 8XS2.

## Supplementary Materials

The following supporting information can be downloaded at: https://www.mdpi.com/article/10.3390/ijms26020648/s1.
