# Supplementary material for: Crystal Structures of Antigen-Binding Fragment of Anti-Osteocalcin Antibody KTM219
Source: Int J Mol Sci. 2025 Jan 14;26(2):648. doi: 10.3390/ijms26020648 (PMC11765575; doi:10.3390/ijms26020648)
Supplement: Supplementary file 1 [file ijms-26-00648-s001.zip › ijms-3379560-supplementary.pdf]

Supplementary Materials

Crystal Structures of Antigen-Binding Fragment of Anti-Osteocalcin Antibody KTM219

Shuma Yazaki [1,†], Misaki Komatsu [1,†], Jinhua Dong [2,‡], Hiroshi Ueda [2], and Ryoichi Arai [1,3,*]

[1] Department of Applied Biology, Faculty of Textile Science and Technology, Shinshu University, Ueda, Nagano 386-8567, Japan

[2] Laboratory for Chemistry and Life Science, Institute of Innovative Research, Tokyo Institute of Technology, Yokohama, Kanagawa 226-8503, Japan

[3] Department of Biomolecular Innovation, Institute for Biomedical Sciences, Interdisciplinary Cluster for Cutting Edge Research, Shinshu University, Ueda, Nagano 386-8567, Japan

*Correspondence: rarai@shinshu-u.ac.jp

†These two authors equally contributed to the work.

‡ Current address:
School of Rehabilitation Sciences and Engineering, University of Health and Rehabilitation Sciences, Qingdao 266071, China

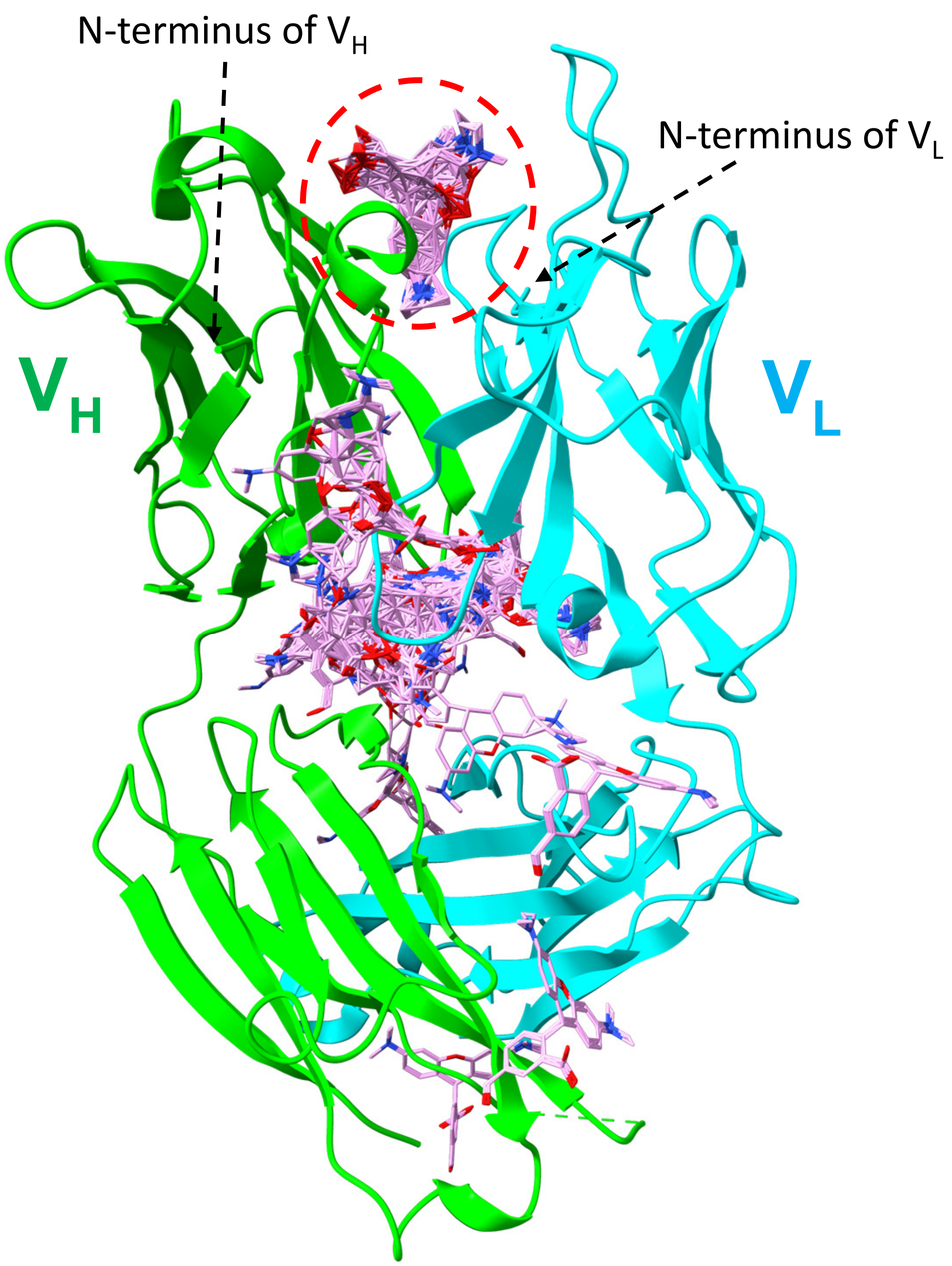

Figure S1. Overall view of docking simulation results of TAMRA to KTM219 Fab using SwissDock [22]. The KTM219 Fab structure of the crystal grown in the presence of TAMRA (PDB ID: 8XS2) was used for docking simulation. Docking poses of TAMRA are shown as pink sticks. Twenty-four poses form dense clusters in the antigen binding pocket as shown by a dotted red circle. We focused on the antigen binding pocket near the N-terminus of V_H as a major interaction site because we focused on the single TAMRA-labeled Q-body (KTM219 Fab fluorolabeled at the N-terminus of V_H) in this study.

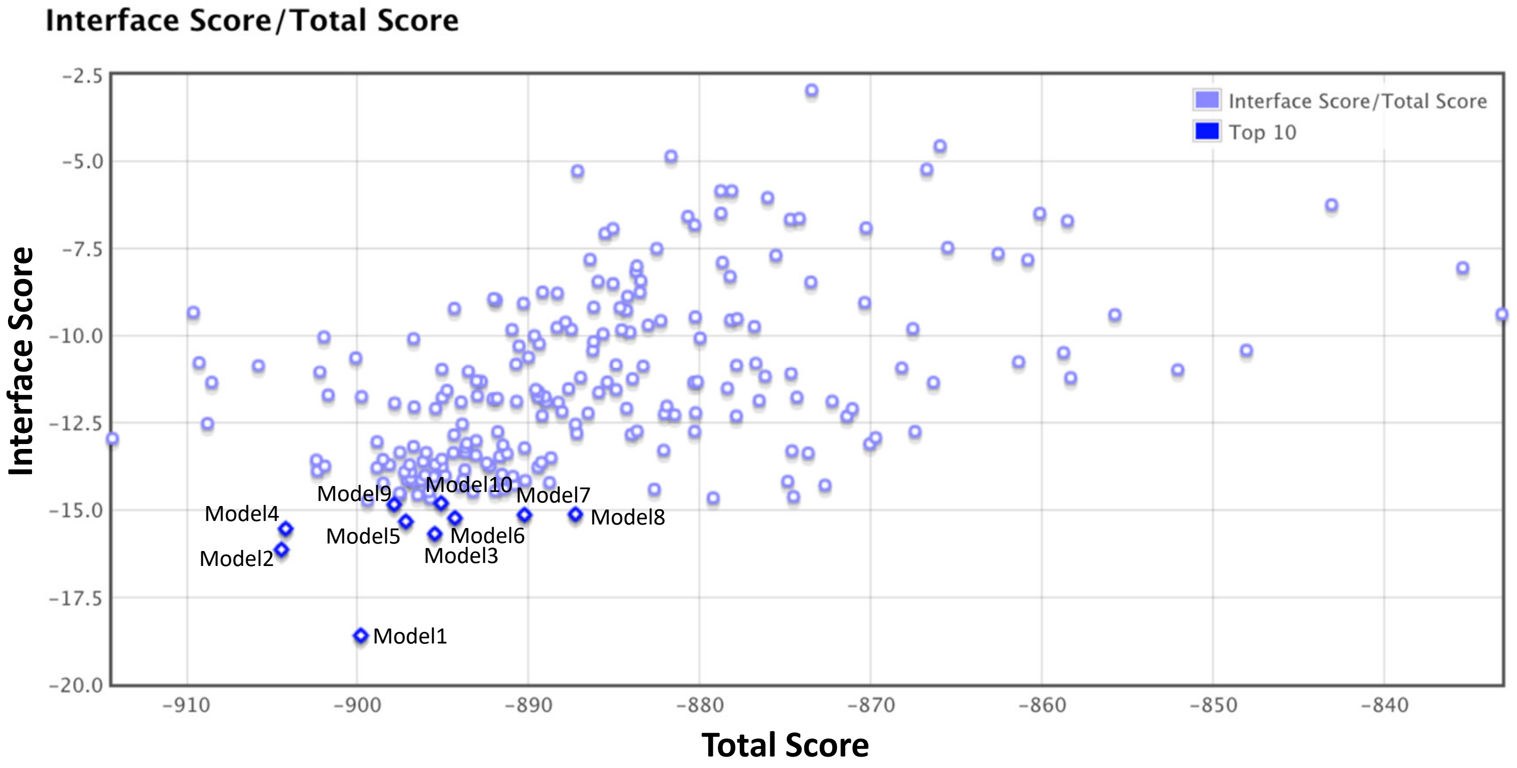

Figure S2. Score plots of docking simulation of TAMRA to KTM219 Fab using RosettaLigand in ROSIE [24]. Interface scores and total scores are plotted on a graph. The top 10 models of interface scores are indicated in the graph. (Lower scores suggest better models.)

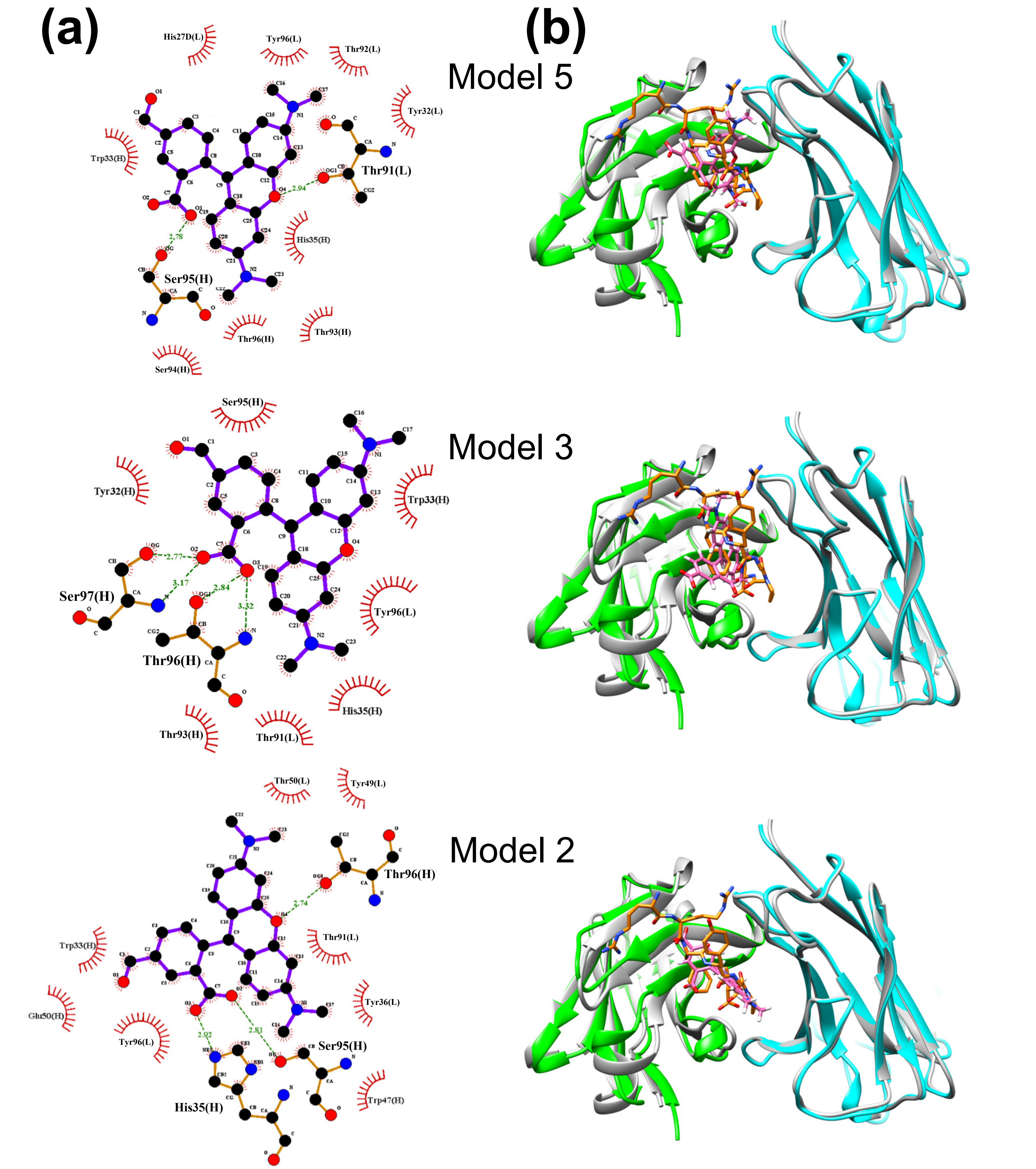

Figure S3. Typical docking simulation results of a fluorescent dye TAMRA to KTM219 Fab using RosettaLigand in ROSIE. (**a**) Interaction diagrams depicted using LigPlot+ [20]. The meaning of the items on the plot is shown in the same key as in Figure 2c. (**b**) Superimpositions of the docking simulation models and the crystal structure of the antibody-antigen complex in the variable region (Fv). The V_H and V_L domains, and TAMRA of the docking models are shown in green, cyan, and pink, respectively. The structures of the antigen (BGP-C7) and Fv in the crystal structure of the antibody-antigen complex (PDB ID: 8XS1) are shown as orange sticks and gray ribbons, respectively. The binding sites of TAMRA and the antigen are significantly overlapped.

Table S1. X-ray data collection and refinement statistics.

	KTM219 Fab	KTM219 Fab + BGP-C7	KTM219 Fab + TAMRA
Data collection			
Space group	$P2_122_1$	$P2_12_12_1$	$P2_122_1$
Unit-cell parameters (Å)	a =64.80<br>b = 71.47<br>c =96.88	a = 43.88,<br>b = 67.49,<br>c =138.66	a =66.19<br>b = 69.52<br>c =96.65
X-ray source	PF BL-5A	PF AR-NW12A	PF BL-5A
Wavelength (Å)	1.00	1.00	1.00
Resolution (Å)	50.0-1.90<br>(1.97-1.90)	50.0-2.30<br>(2.38-2.30)	50.0-2.14<br>(2.18-2.14)
Unique reflections	35733	18912	24972
Average redundancy	6.8 (6.8)	6.1 (6.5)	12.6 (11.8)
Completeness (%)	98.8 (97.5)	99.1 (99.6)	99.5 (98.1)
I / σ(I)	12.5 (4.23)	5.30 (2.59)	5.40 (2.47)
R_{meas} (%)	7.1 (52.2)	16.5 (83.5)	12.0 (106.0)
$CC_{1/2}$	(0.918)	(0.836)	(0.815)
Refinement			
Resolution (Å)	50.0-1.90	50.0-2.30	50.0-2.14
No. of reflections	33891	17875	23209
No. of protein atoms	3271	3319	3237
No. of water atoms	239	128	257
No. of other atoms	83	0	8
R_{work} (%)	18.1	19.9	20.2
R_{free} (%)[1]	22.4	24.8	24.4
RMSD bond length (Å)	0.011	0.011	0.010
RMSD bond angles (˚)	1.54	1.65	1.46
Average B-factor (Å^2)	31.0	39.5	30.0
Ramachandran plot[2]			
In preferred regions (%)	97.9	95.4	97.2
In allowed regions (%)	2.1	4.6	2.8
Outliers (%)	0	0	0
PDB ID	5X5X	8XS1	8XS2

All numbers in parentheses represent last outer shell statistics.

[1]R_{free} is calculated for 5% of randomly selected reflections excluded from refinement.

[2] Ramachandran plot analyses of the models were performed using RAMPAGE [32].

Table S2. Distances between Trp residues and TAMRA in the results of RosettaLigand docking simulation.

	Trp33(H)	Trp36(H)	Trp47(H)	Trp103(H)	Trp35(L)
Model 2	7.9 Å	15.3 Å	10.2 Å	12.4 Å	16.0 Å
Model 3	5.2 Å	14.0 Å	9.9 Å	12.5 Å	17.7 Å
Model 5	6.3 Å	14.6 Å	9.6 Å	12.7 Å	17.3 Å
Model 6	6.0 Å	14.0 Å	10.1 Å	11.1 Å	17.4 Å
Model 7	6.4 Å	14.6 Å	9.5 Å	12.8 Å	17.3 Å
Model 8	5.6 Å	15.7 Å	10.4 Å	13.2 Å	16.8 Å
Model 9	5.6 Å	16.9 Å	11.2 Å	16.7 Å	20.6 Å
Model 10	6.1 Å	14.5 Å	9.6 Å	12.8 Å	17.5 Å

Distances between CD2 atom of Trp and C01 atom of TAMRA (see diagram below) were measured.

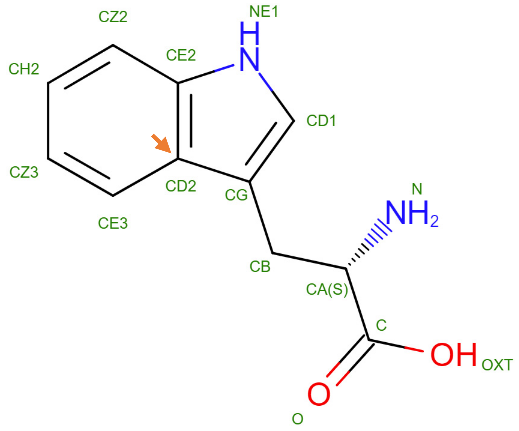

Tryptophan (Trp)

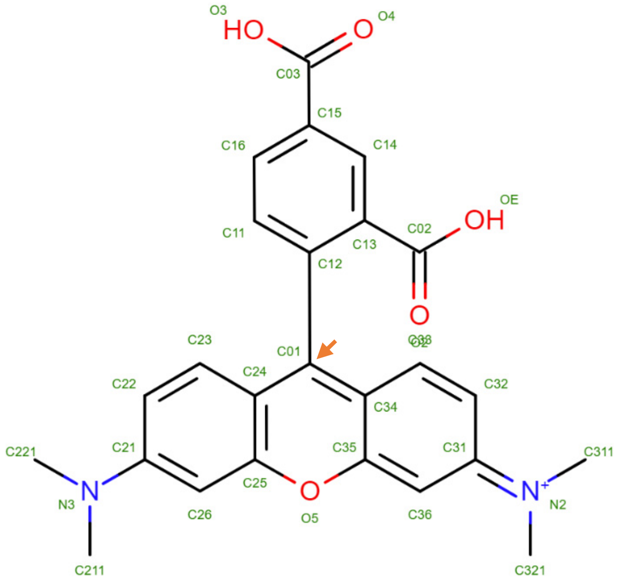

5-carboxytetramethylrhodamine (TAMRA)
